# Determination of Serum Arginase-1 Concentrations and Serum Arginase Activity for the Non-Invasive Diagnosis of Endometriosis

**DOI:** 10.3390/jcm13051489

**Published:** 2024-03-05

**Authors:** Maciej Pliszkiewicz, Malgorzata Czystowska-Kuzmicz, Karolina Soroczynska, Bogumił Paweł Siekierski, Krzysztof Safranow

**Affiliations:** 1 Department of Gynaecology, Medicover Hospital, 02-972 Warsaw, Poland; 2 Chair and Department of Biochemistry, Medical University of Warsaw, Banacha 1 St., 02-097 Warsaw, Poland; 3 Department of Biochemistry and Medical Chemistry, Pomeranian Medical University in Szczecin, Powstańców Wlkp. 72, 70-111 Szczecin, Poland

**Keywords:** endometriosis, endometriosis diagnosis, arginase, non-invasive, biomarker, immunosuppression

## Abstract

**Backgroud:** Endometriosis remains a diagnostic challenge, both clinically and economically, affecting 6% to 15% of women of child-bearing potential. We have attempted to determine whether testing serum concentrations and activity of arginase isoenzymes could be useful for the non-invasive diagnosis of endometriosis. **Methods:** This study involved 180 women (105 endometriosis subjects—study group B; 22 subjects with other benign gynaecological conditions—control group 1—K1, both undergoing surgery; and 53 healthy subjects without features of endometriosis—control group 2—K2). **Results:** Preoperative and postoperative arginase-1 (Arg-1) concentrations were significantly higher in patients, as compared with the control groups K1 (*p* < 0.0001 and *p* = 0.0005, respectively) and K2 (both *p* < 0.0001). Similarly, arginase activity was significantly higher in patients than in the control group K1 before surgery and higher than in both control groups after surgery. No significant differences in either Arg-1 concentrations or arginase activity were noted between the operated control group K1 and the non-operated control group K2. A significant postoperative decrease in Arg-1 concentration was observed within both patient (*p* < 0.0001) and control group K1 (*p* = 0.0043). Diagnostic performance was assessed using the receiver operating characteristic (ROC) method. The threshold for differentiation between endometriosis patients and healthy non-operated controls was 42.3 ng/mL, with a sensitivity of 90% and specificity of 81%. For differentiation of patients and operated controls with benign gynaecological conditions, the threshold was 78.4 ng/mL, with a sensitivity of 61% and specificity of 95%. **Conclusions:** We, therefore, conclude that Arg-1 serum concentrations and arginase activity could be considered potential biomarkers for endometriosis but require further studies on larger cohorts of patients.

## 1. Introduction

Endometriosis is defined as the occurrence and proliferation of tissue with histological characteristics of the uterine lining (endometrium) outside the uterine cavity (ectopically) [1]. The biology of the disease, its mode of spreading, the ability to damage the structure and function of adjacent organs, as well as challenges related to its diagnosis and treatment, make it resemble a proliferative-type disease, except for what distinguishes it from malignancies—it is not a life-threatening condition. However, it leaves no lesser imprint on the daily lives of women affected by the disease. Intense, cyclical, or persistent pain symptoms, such as dyspareunia or chronic, treatment-resistant infertility, often become the cause of repeated work absences and problems fulfilling social roles, especially in relationships [2].

Depending on sources, it is estimated that endometriosis occurs in 6 to 15% of women of child-bearing potential. This is due to delayed diagnosis and low awareness among both patients and medical staff. It is believed that up to 190 million women worldwide suffer from this condition [3].

The etiopathogenesis of this oestrogen-dependent disorder has not been fully explained. There is no integrated theory explaining the development of the disease that would allow a causal treatment to be designed and implemented. Proposed theories of endometriosis development can be divided into three groups, depending on how lesions are believed to emerge. These are in situ development theories, induction theories, transplantation theories, and genetics-based theories [4].

The dysfunction of the immune system may also indirectly induce the formation and proliferation of endometrial lesions. Increased levels of activated macrophages, together with impaired cell-mediated immunity and depleted natural killer (NK) cell function, are noted in both bloodstream and peritoneal fluid. This may lead to reduced efficacy of immune surveillance, impairing processes of clearing endometrial remnants from the peritoneum, thus facilitating ectopic implantation and growth of endometrioid cells [5]. Survival and resistance of endometrioid cells to lysis by immune system cells result from the ability of these to “mask” themselves and evade the immune system by modulating, for example, the expression of HLA class I antigens in ectopic endometrioid cells [6].

### 1.1. Diagnostics

The diagnosis of endometriosis is complex, as evidenced by the average time from onset of symptoms to final diagnosis of the disease, which averages up to 9 years. The primary diagnostic tools include thorough medical history and clinical examination, supplemented by imaging modalities. Methods for laboratory verification of the diagnosis are still being sought.

#### 1.1.1. Imaging

Ultrasonography (USG) is the first-line diagnostic method owing to its widespread availability and relatively low cost. Since the development of the IDEA protocol (International Deep Endometriosis Analysis) [7], it has become a basic tool for the diagnosis of suspected endometriosis. Should ultrasound images remain unclear, it is recommended that the next step of examination be performed, namely magnetic resonance imaging (MRI) [8]. Until 2022 and the publishing of new European Society of Human Reproduction and Embryology (ESHRE) guidelines for the management of endometriosis [9], diagnostic laparoscopy, preferably combined with histopathology material sampling, was the “gold standard” in the diagnosis of endometriosis [10]. Currently, performing diagnostic laparoscopy alone is no longer recommended [11]. Other imaging modalities are rarely used in the diagnosis of the disease.

#### 1.1.2. Laboratory Diagnostic Methods

Extensive research has been undertaken to develop a diagnostic test to replace invasive diagnostic procedures like diagnostic laparoscopy with histopathological examination following an unclear first-line ultrasound assessment.

Extensive systematic reviews have been conducted to identify substances present in blood urine or sampled invasively from the uterine cavity, which could meet the criteria of a screening test [12,13,14]. However, so far, none of these meta-analyses has yielded any non-invasive marker for which the diagnostic performance would have reached the replacement test criteria (sensitivity ≥ 94% and specificity ≥ 79%) or screening test criteria, with a sensitivity ≥ 95% and specificity ≥ 50% (SnOUT), or a sensitivity ≥ 50% with specificity ≥ 95% (SpIN) [12,15]. There are new and promising reports regarding the use of miRNA [16,17] or small molecule metabolites [18] as potential markers for endometriosis, but there are still no studies validating their use in a clinical setting.

### 1.2. L-Arginine and Its Significance in Immune Response

L-arginine (2-amino-5-guanidinopentanoic acid) is a semi-essential α-amino acid, synthesised primarily in the liver and kidneys. L-arginine produced in the urea cycle within the liver is not released into the bloodstream because it is metabolised by the urea cycle hydrolase, arginase-1 (Arg-1), into L-citrulline and urea. Approximately 60% of endogenous L-arginine is produced from citrulline in the proximal renal tubules, involving argininosuccinate synthase and argininosuccinate lyase [19]. Endothelial cells are another site of L-arginine production, where L-arginine is formed in a mechanism similar to that within the kidneys [20].

L-arginine has a significant impact on the immune system function—it is essential for the activation and proliferation of T-cells [21,22] and, consequently, for initiating an effective immune response (including antitumor response). Its deficiency or absence in the environment leads to the inhibition of T-cell proliferation, which results in a decreased destruction of foreign cells (e.g., cancer cells) [23]. Moreover, under L-arginine depletion, T-cells show reduced expression of the CD3ζ chain, a crucial component of the T-cell receptor (TCR) complex involved in signalling and T-cell activation [24], as well as impaired cytokine production (e.g., of IFN-γ).

At the same time, L-arginine is an essential substrate for the development of some cancer cells. Therefore, a dilemma arises—is it therapeutically beneficial to reduce the availability of L-arginine to potentially inhibit tumour cell growth, or, on the contrary, is it better to increase its availability to enhance the immune response [25]? There are no data regarding a potential relationship between endometriosis growth and L-arginine bioavailability.

### 1.3. Arginase and Its Importance in Pathology

Arginase is an Mn^2+^-dependent hydrolase with two isoforms, Arg-1 and arginase-2 (Arg-2), encoded by separate genes located on different chromosomes (chromosome 6 (6q23) and chromosome 14 (14q24.1-24.3), respectively). Both isoenzymes occur as homotrimers and have an identical mechanism of action involving the cleavage of L-arginine into L-ornithine and urea. They exhibit approximately 60% amino acid homology, with 100% homologous enzymatic activity sites [26].

Arg-1 (also known as cytosolic or hepatic form) shows the highest expression in hepatocytes, where it participates primarily in nitrogen metabolism, being the final component of the urea cycle [26]. Usually, it is not released into the bloodstream, except in situations of liver damage—studies indicate a significant negative correlation between arginase activity and liver function [27], e.g., in liver transplant recipients with failed treatment [28]. Arg-1 is also present in erythrocytes and certain bone myeloid cells (such as macrophages). High expression of Arg-1 has been demonstrated in myeloid-derived suppressor cells (MDSCs) in many types of cancer, inhibiting the activity of T-cells [29]. The half-life of Arg-1 in the blood is not clearly defined, as the available literature provides information varying between below 30 min and up to 5 h [30].

Arg-2 (also known as mitochondrial or extrahepatic) shows the highest expression in the kidneys but is also present in the thyroid and prostate in men, although it is found in most extrahepatic tissues. Its role is not yet well established. The half-life of Arg-2 in the bloodstream under normoxic conditions is estimated to be approximately 12 h [31].

Dysregulated expression and activity of Arg-1 and/or Arg-2 may have significant implications in the pathogenesis and progression of a wide range of pathologies, including cardiovascular and neurological diseases and a wide range of malignancies [32].

Elevated arginase levels, associated with adverse outcomes in various diseases, remain largely unexplored in gynaecological conditions, especially endometriosis. However, emerging evidence highlights its significance in cervical, ovarian, and endometrial cancers, offering promising avenues for diagnostic and therapeutic advancements.

Clinical studies in cervical cancer reveal a substantial increase in arginase activity, particularly in women with squamous cell carcinoma (SCC) and advanced-grade lesions, suggesting its potential as a diagnostic marker [33]. The upregulation of Arg-1 expression in cervical tissue samples, along with lesion severity and M2 macrophage density, emphasised its role in disease progression [34]. Another investigation in patients with abnormal cytology shows a significant correlation between arginase activity and cervical lesion development. Arg-1 was implicated in HPV infection persistence and precancerous lesion promotion, while Arg-2 appeared to be involved in the progression to invasive cervical cancer [35].

In endometrial cancer, arginase was significantly elevated in patients compared to healthy controls, suggesting its potential as a diagnostic marker [36]. This was confirmed by the group of Tran et al., who identified Arg-1 as a potential prognostic marker in metastatic and recurrent endometrial cancer (EC). They observed increased expression in early-stage EC and endometrial hyperplasia from mice deficient in Mig-6 and Pten mutations, indicating an association with poor prognosis [37].

Moreover, elevated arginase activity in patients’ plasma, decreasing post-chemotherapy, suggests its potential as a dynamic biomarker for ovarian cancer (OvCa) [38]. Arg-1 produced in vascular leukocytes within peritoneal ovarian tumours was shown to contribute to immune suppression, hindering T-cell responses [39]. It is consistent with the findings of another study that showed that high Arg-1 expression in primary tumours and increased activity in plasma correlated with a worse prognosis. The authors revealed that OvCa cells release Arg-1 in small extracellular vesicles (EVs), which mitigated anti-tumour immune responses by inhibiting T-cell activation and proliferation. Blocking arginase activity mitigated Arg-1-driven tumour progression, providing a potential therapeutic target [40].

### 1.4. Role of Arginine and Arginase in Endometriosis–Hypothesis

A similar, however temporary, down-regulation of T-cell activity by Arg-1 has been observed in the female reproductive tract during the menstrual cycle and normal pregnancy. It has been shown that through the transient synchronous upregulation of Arg-1 and IDO during the secretory phase of the menstrual cycle, the cytolytic activity of CD8 T-cells is downregulated to provide specific immune tolerance conditions for conception and later also for pregnancy [41]. Also, endometriosis shares many common features with cancers, such as the mode of spreading, infiltration, and destruction of tissue and organ structures, as well as the potential for initiating distant lymphatic or bloodborne metastasis. Endometriotic lesions, being abnormal implants of endometrioid tissue, also evade immune surveillance to some extent, and similar mechanisms as in cancer pre-metastatic niche formation are believed to play a role in the implementation and growth of ectopic endometrial cells [42,43]. Therefore, we hypothesise that a similar immunosuppressive mechanism mediated by Arg-1, as described in OvCa, may play a role in endometriosis. There is evidence suggesting that the immune tolerance of the uterine cavity during pregnancy, similar to in several cancers, is mediated by the presence of Arg-1-producing MDSCs [44]. Likewise, elevated levels of MDSCs have been reported in endometriosis and linked to similar mechanisms of immune escape [21].

As in OvCa, we assume that elevated levels and activity of Arg-1 and/or Arg-1 may be present in the sera of endometriosis patients and may have diagnostic or prognostic potential. So far, there are no publications about the role of arginase 1 in the endometriosis diagnosis.

### 1.5. Objectives

The objective of this study was to assess the enzymatic activity of arginase and the concentrations of its isoforms, Arg-1 and Arg-2, in the serum of patients with confirmed endometriosis before and after radical conservative (meaning maximum radicality towards the disease but conservatism in terms of preserved fertility) laparoscopic treatment, compared with patients with other benign pelvic pathologies and healthy individuals. The evaluation of differences in arginase concentrations and activity before and after surgical treatment, as well as the comparison of these results to values obtained in the group of patients without endometriosis, could help to determine whether these parameters could be useful diagnostic markers for the diagnosis, staging, and follow-up of endometriosis.

## 2. Materials and Methods

### 2.1. Patients

Following informed consent of the patients (approved by the Bioethical Committee at the Medical University of Warsaw–KB/34/2016 dated 16 February 2016), 180 women were included in this study, including 105 subjects with confirmed endometriosis (study group—B), 22 patients with other benign pelvic pathologies, excluding endometriosis (control group 1—K1), and 53 healthy women (control group 2—K2).

Blood samples were collected to determine the concentrations of Arg-1 and Arg-2, as well as arginase activity, before and after surgery for patients undergoing surgery and once for healthy women at the time of diagnosis. Due to the significant role of the kidneys in arginine metabolism, serum creatinine levels, as well as glucose levels, were determined in all subjects to exclude diabetes.

All endometriosis surgeries were performed by laparoscopy. In the study group, 94% of the procedures were performed by a single surgical team (including the author of this study), ensuring a high level of surgical consistency.

#### 2.1.1. Study Group GB

The study group consisted of 105 women aged 21 to 48 years (median age 34 years) who underwent radical conservative surgical treatment for endometriosis.

Inclusion criteria included age between 18 and 50 years, confirmed endometriosis in previous histopathological examinations (for previously operated patients) or clinically suspected endometriosis (based on history, symptoms, physical examination, and/or imaging), no history or current diagnosis of malignancy, no history or current diagnosis of other significant pathologies that could affect the results (e.g., kidney diseases, connective tissue diseases, diabetes), consent to participate in the study and consent for suggested surgical treatment. Exclusion criteria included age < 18 or >50 years, confirmed postmenopausal status, history or current diagnosis of malignancy, presence of other significant pathologies that could affect the results, and refusal of participation in the study or treatment.

The distribution by disease severity and surgery outcome within the study group is presented in Supplementary Table S1. For patients undergoing surgery, blood samples were collected on the day before surgery and 48 h after surgery. The choice of postoperative sampling time resulted from logistical considerations. In the postoperative period, patients in group B were observed for any recurrence/progression of the disease, defined as postoperative recurrence or exacerbation of existing symptoms and/or the recurrence of endometriosis-like imaging evidence. During this period, a total of 15 recurrences (14% of patients) were recorded (Supplementary Table S2). Within the study group, 45 patients were treated for endometriosis and associated infertility. All of them subsequently actively attempted to conceive, and of these, 31 conceived (with 5 patients conceiving following ART methods. Of those 14 patients who did not conceive, 3 received ART treatment.

#### 2.1.2. Control Groups

The control group K1 included 22 women aged 19 to 49 years (median age 40 years) who underwent surgery for benign gynaecological conditions other than endometriosis. Inclusion criteria were: age range of 18–50 years, no data indicating the presence of endometriosis (data from previous surgeries, if performed, history, symptoms, clinical features, including physical and/or imaging examination), no history or current diagnosis of malignancy, no history or current diagnosis of other significant pathologies that could affect the results (e.g., kidney diseases, connective tissue diseases, diabetes), consent to participate in the study and for the suggested surgical treatment. Exclusion criteria were the same as for the patient group. Indications for surgery of these patients are presented in Supplementary Table S3. Blood samples were also planned to be collected before and after surgery in these patients, following the same timeline as for the study group patients).

The control group K2 consisted of healthy women with no history, symptoms, or other clinical features of endometriosis, as well as no history or current diagnosis of malignancy and no history or current diagnosis of other significant pathologies that could affect the results. Informed consent to participate in this study was also required. Women with autoimmune diseases (such as Hashimoto’s disease), potentially affecting the assessed parameters, were also excluded from this group. Ultimately, 53 women aged 18 to 48 years (median age 34 years) were included in the K2 group, with a single blood sample collected to determine serum levels of Arg-1, Arg-2, and arginase activity, as well as blood glucose and creatinine levels.

Detailed characteristics of the analysed study and control groups according to age, fasting blood glucose, and serum levels are listed under Supplementary Materials (Supplementary Tables S3 and S4 and Figure S1).

### 2.2. Serum Preparation

After collection, blood was left to clot under refrigerated conditions for 1–2 h, followed by centrifugation for 15 min at 3500 rpm (equivalent to 1500× *g*). The obtained sera were transferred to code-labelled polypropylene tubes (two per collection) and frozen at −30 °C. Serum samples showing signs of haemolysis were discarded before the freezing step due to the high Arg-1 content in erythrocytes, which could significantly interfere with the assay results. Subsequently, the frozen serum samples were transported to the laboratory and stored at −80 °C until the assays were performed.

### 2.3. Arg-1 and Arg-2 ELISA and Arginase Activity

Concentrations of Arg-1 and Arg-2 isoenzymes were determined using dedicated enzyme-linked immunosorbent assay (ELISA) kits, according to the instructions of the providers. For Arg-1 measurements, Hycult^®^ Biotech’s human Arginase I assay kit (Uden, The Netherlands; Catalogue No. HK386-01) was used, with a detection range of 1.6–100 ng/mL, and for Arg-2 measurements, Aviva Systems Biology’s human Arginase II assay kit (San Diego, CA, USA; Catalogue No. OKCD01118) was utilised, with a detection range of 3.12–200 ng/mL. Each sample was measured twice.

For the determination of total arginase activity (without differentiating between Arg-1 and Arg-2 isoenzymes), SigmaAldrich^®^ Arginase Activity Assay Kit (St. Louis, MO, USA; Catalogue No. MAK112) was employed, following the protocol of the provider. Technical repetitions were measured for each sample. All measurements were performed on the Asys UVM 340 Microplate Reader (Biochrom, Cambridge, UK) spectrophotometer. Blood glucose and creatinine measurements were performed in certified medical diagnostic laboratories.

### 2.4. Statistical Analysis

Statistical analysis included descriptive statistics such as mean, standard deviation, median, interquartile range (lower–upper quartile), and range (minimum–maximum) for measurable variables. The normality of the distribution of measurable variables was assessed using the Shapiro–Wilk test. Since the distributions of most variables, including Arg-1 concentration and arginase activity, significantly deviated from the normal distribution, non-parametric tests were used for their analysis: the Mann–Whitney U test for comparisons between groups, the Wilcoxon signed-rank test for paired comparisons of results before and after surgery, and the Spearman rank correlation coefficient for assessing the significance of relationships between different measurable and rank parameters.

The association of arginase concentrations and activity with the risk of endometriosis recurrence and the chance of getting pregnant after surgery was analysed using the Cox proportional hazards model. The diagnostic value of arginase concentrations and activity in relation to the diagnosis of endometriosis was analysed using Receiver Operating Characteristics (ROC) techniques, determining proposed cutoff points based on the Youden index. Sensitivity, specificity, and the area under the ROC curve (AUC) were calculated with standard error (SE) and a 95% confidence interval (95% CI). The significance threshold was set at *p* < 0.05. Only statistically significant differences are marked in the figures presented below. Statistical calculations were performed using the Statistica version 13 with the Plus Add-on.

## 3. Results

### 3.1. Evaluation of Assay Quality

The measurements of Arg-2 concentration in serum could not be considered reliable. In repeated assays of Arg-2 for 16 random samples in groups GB and K2, a lack of assay reproducibility was observed (up to threefold differences in Arg-2 concentration values for the same serum sample analysed twice in separate assay series) despite the rigorous application of identical analytical procedures. Therefore, these measurements were excluded from further statistical analysis in this study. No issues were observed regarding the reproducibility of measurements for Arg-1 concentrations or arginase activity.

### 3.2. Evaluation of Preoperative Serum Arginase Levels and Activity

**Figure 1:**
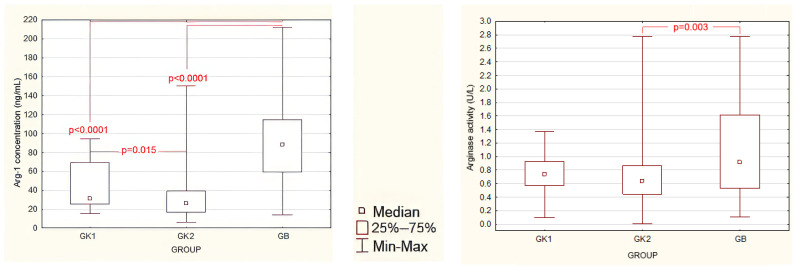
Preoperative serum Arg-1 concentrations (**left panel**) and serum arginase activity (**right panel**) in study groups: GB—study group; GK1—control group 1; GK2—control group 2. Statistically significant differences are marked (Mann–Whitney U test).

In preoperative assessments, Arg-1 concentrations were statistically significantly higher in patients compared to both control groups K1 and K2 (Figure 1 left panel, Supplementary Table S5). Furthermore, it was observed that in the K2 group of healthy women, Arg-1 concentration was significantly lower than in the K1 group of women operated for reasons other than endometriosis.

Before surgery, arginase activity values were statistically significantly higher in patients compared to group K2. However, no significant differences in arginase enzymatic activity were found between patients and group K1 as well as between both control groups (Figure 1 right panel and Supplementary Table S5).

### 3.3. Evaluation of Postoperative Serum Arginase Concentrations and Activities

**Figure 2:**
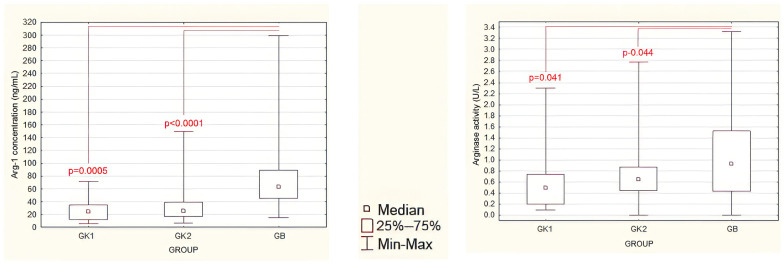
Postoperative serum Arg-1 concentrations (**left panel**) and serum arginase activity (**right panel**) in operated groups: GB—study group; GK1—control group 1; and in the non-operated GK2—control group 2. Statistically significant differences are marked (Mann–Whitney U test).

Postoperative Arg-1 concentrations in patients were significantly higher compared to K1 and K2 controls (Figure 2 left panel, Supplementary Table S6). The same was true for arginase activity after surgery (Figure 2 right panel, Supplementary Table S6), which was significantly higher in group GB compared to groups K1 and K2, although these associations had a lower level of statistical significance than Arg-1 concentration. There were no significant differences in Arg-1 concentration or arginase activity between group K1 after surgery and the non-operated group K2.

### 3.4. Evaluation of Perioperative Serum Arg-1 Concentration Changes

**Figure 3:**
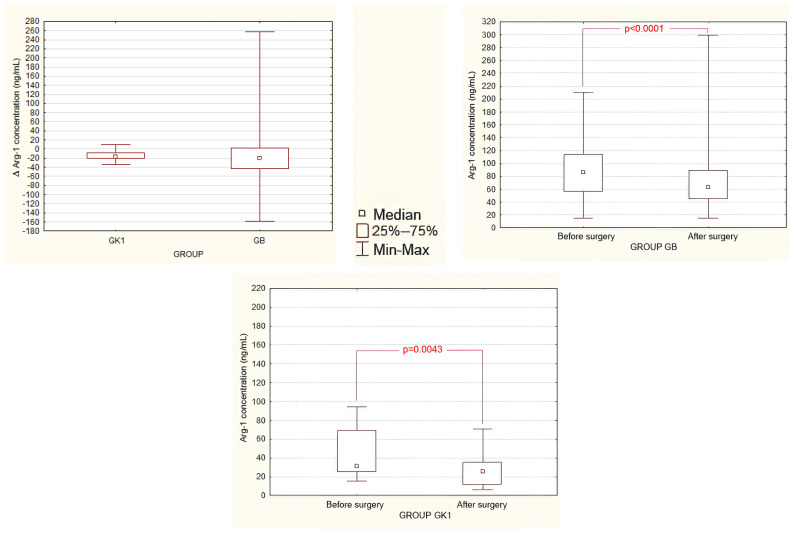
Serum Arg-1 concentrations before and after surgery. Perioperative change (Δ) in serum Arg-1 concentrations in groups B and K1 (**upper left panel**). Comparison of serum Arg-1 concentrations in the study group GB (**upper right panel**) and the control GK1 group (**lower panel**) before and after surgery. The statistically significant difference is marked (Wilcoxon pair signed-rank test).

A statistically significant decrease in the concentration of Arg-1 after surgery was observed in both patients group GB and group K1 (Figure 3 and Supplementary Table S7). However, there was no significant difference in the change in Arg-1 concentration measured before and after surgery between group GB and group K1.

### 3.5. Evaluation of Perioperative Serum Arginase Activity Changes

There was no statistically significant change in arginase activity after surgery compared to the preoperative values in both group GB and group K1. Additionally, no significant difference in the change in arginase activity between groups GB and K1 was observed (Supplementary Table S8 and Figures S2–S6).

### 3.6. Analysis of Sensitivity and Specificity of Measurements of Arginase Concentrations and Activity in the Diagnosis of Endometriosis

ROC analysis of the sensitivity and specificity was performed to evaluate the potential of serum Arg-1 concentration and arginase activity as diagnostic indicators of endometriosis.

**Figure 4:**
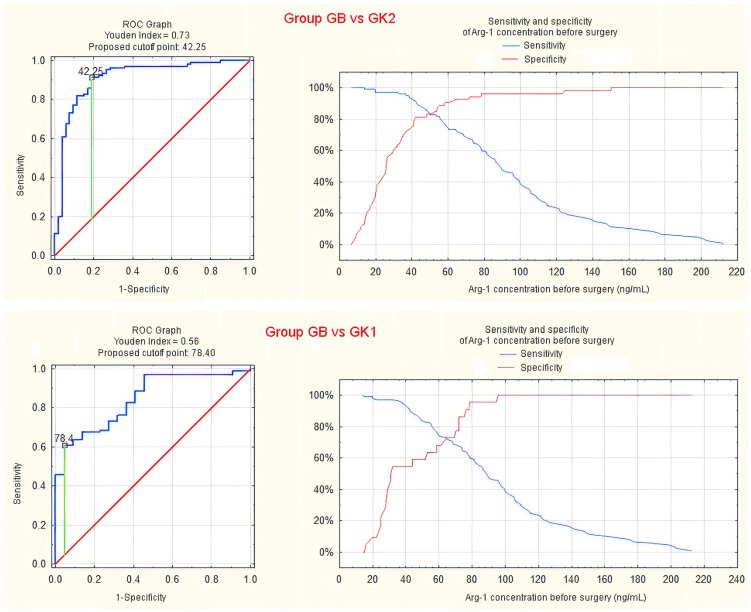
ROC curve and sensitivity/specificity graph for preoperative serum Arg-1 concentration differentiating groups GB and GK2 (**upper panel**) or GK1 (**lower panel**).

High Arg-1 concentration effectively differentiated group GB from group K2 and, to a lesser extent, from group K1 (Figure 4 and Table 1). The proposed cutoff based on the Youden index for discriminating between groups GB and K2 had a value of 42.3 ng/mL, providing a sensitivity of 90% and specificity of 81%. The cutoff for discriminating between groups GB and K1 was 78.4 ng/mL, providing a sensitivity of 61% and specificity of 95%.

Applying the same method for assessing the test utility [12], the serum Arg-1 concentration reached the threshold criteria for replacement test discriminating endometriosis patients from healthy controls—showing sensitivity of 94.2% and specificity of 73.6% with a cutoff for Arg-1 concentration of 38.5 ng/ (meeting the criteria for sensitivity ≥ 94% and specificity ≥ 79%). It fulfils both the SnOUT (high SeNsitivity to rule OUT—a negative result rules out the disease)—sensitivity of 95.2% with a specificity of 73.6% for a cutoff Arg-1 concentration of 38.15 ng/mL (meeting the criteria for sensitivity ≥ 95% and specificity ≥ 50%) and the SpIN (high SPecificity to rule IN—a positive result indicates the presence of the disease)—sensitivity of 60.9% with a specificity of 96.2% for a cutoff Arg-1 concentration of 78.4 ng/mL (meeting the criteria for sensitivity ≥ 50% and specificity ≥ 95%).

**Figure 5:**
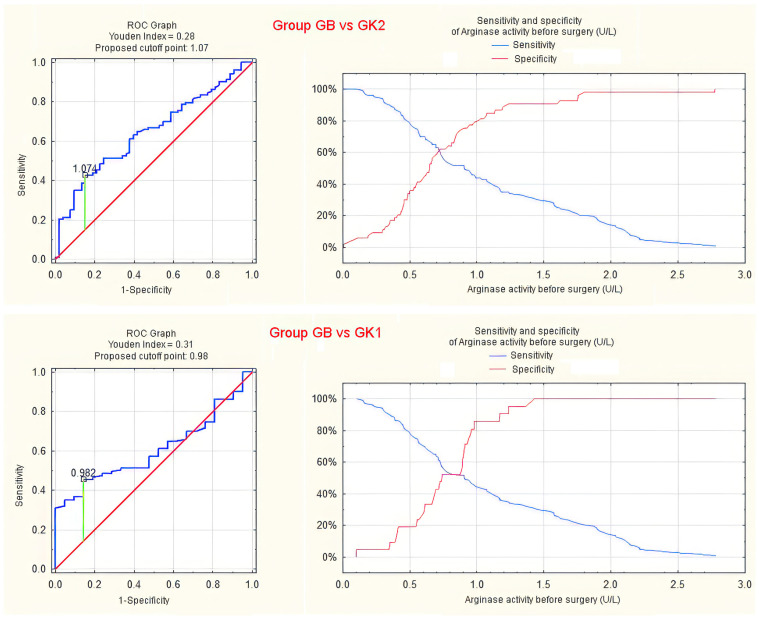
ROC curve and sensitivity/specificity graph for preoperative serum arginase activity differentiating groups GB and GK2 (**upper panel**) or GK1 (**lower panel**).

In the case of arginase activity, statistical significance was also demonstrated for differentiating group GB from groups K1 and K2. However, considering the shape of the ROC curve and the small area under the curve (AUC < 0.7), the diagnostic value should be considered quite low and not clinically useful (Figure 5 and Supplementary Table S9).

### 3.7. Correlations

**Figure 6:**
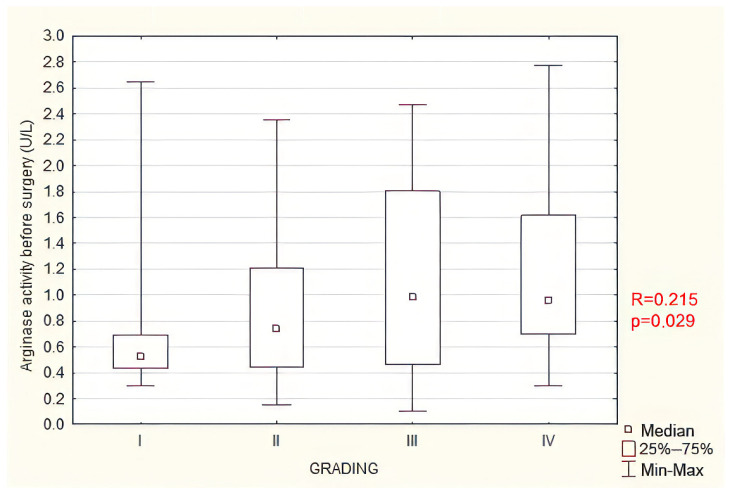
Correlation between preoperative serum arginase activity and clinical severity of endometriosis (ASRM grading). R—Spearman rank correlation test.

A statistically significant positive, however weak correlation was found between preoperative arginase activity values and the clinical severity of endometriosis according to the ASRM scale (Figure 6). No significant relationship was observed between the clinical severity and preoperative serum Arg-1 levels. There were also statistically significant positive, however weak correlations between preoperative arginase activity and preoperative serum Arg-1 levels (Supplementary Figure S5), between fasting blood glucose levels and preoperative (Supplementary Figure S6) or postoperative arginase activity (Supplementary Figure S7) as well as between the change in Arg-1 concentration (Δ ARG1) and the change in arginase activity (Δ ARGACT, Supplementary Figure S8). The detailed correlations are listed in Supplementary Tables and Figures.

### 3.8. Recurrence Risk and Chance of Achieving Pregnancy

Recurrence or progression of the disease in the postoperative period was observed in a total of 15 patients out of 105 in group GB (14%). The risk of recurrence was assessed using a univariate Cox proportional hazards model. The analysis of the measured pre- and postoperative Arg-1 parameters did not show a statistically significant association between these parameters and the risk of endometriosis recurrence after surgical treatment (Supplementary Table S12).

The chance of achieving pregnancy in the postoperative period was observed in a group of 45 women with infertility, who all made active attempts to conceive. Of these, pregnancy was achieved in 31 patients (69%, with five patients conceiving following ART methods) during the observation period (Supplementary Table S13). The assessment of the chance of achieving pregnancy was conducted using survival analysis according to the Cox proportional hazards model. The analysis did not show a statistically significant association between these parameters and the likelihood of achieving pregnancy after surgical treatment of endometriosis (Supplementary Table S14).

## 4. Discussion

### 4.1. Diagnostic Value of Arginase Concentration and Activity Measurements in Serum for the Diagnosis of Endometriosis

We have shown that the determination of Arg-1 concentration could be considered a promising test for differentiating patients with endometriosis from patients with other gynaecological disorders (ROC AUC = 0.848 for groups GB and K1). The lower limit of the 95% confidence interval (0.769) indicates that in the worst case, the diagnostic value of such a test will not be worse than average. Furthermore, we have shown that the diagnostic value of Arg-1 serum concentration was even higher in differentiating patients with endometriosis from healthy, asymptomatic women (ROC AUC = 0.912, lower limit of the 95% confidence interval = 0.859 for groups B and K2).

Analysis of differences in arginase activity between patients and controls, despite achieving statistical significance, does not indicate any diagnostic usefulness of a potential test (ROC AUC = 0.608 for GB compared to GK1 and 0.646 for GB compared to GK2, with a lower limit of the 95% confidence interval of 0.501 and 0.559, respectively).

The use of Arg-1 concentration determination as a diagnostic test could have a practical supportive value in women with clinical suspicion of endometriosis but without clear imaging features (such as endometrial cysts). In the case of other benign gynaecological pathologies, the differentiating value of the test might be lower due to the lower sensitivity and specificity values obtained in our analysis when comparing endometriosis patients with the benign control group K1.

### 4.2. Limitations of This Study

It has to be noted that neither of the two chosen control groups in this study constitutes an ideal control group in regard to endometriosis patients. The ideal control group would be a population of women selected based on demographic criteria, presenting symptoms that may be suggestive of endometriosis (such as dysmenorrhoea) but with confirmed absence of this condition.

Regarding postoperative determinations, the optimal time interval from surgery to potential postoperative determination of Arg-1 concentration and arginase activity remains undefined. The 48 h interval adopted in this study, dictated by logistical considerations, may indeed prove to be too short for a reliable assessment of obtained values and correlations. Considering that the half-life of arginase isoenzymes, which can be up to about 12 h, according to the literature, the mentioned 48 h period may be insufficient to draw clear conclusions about the impact of the surgical procedure on postoperative Arg-1 concentrations and arginase activity. In this regard, it would be worth assessing the above parameters after a longer period, e.g., 7 days or even a month (to also limit the potential impact of the inflammation associated with surgical trauma and healing processes). Therefore, the results of this study do not allow for clear conclusions about the usefulness of postoperative Arg-1 concentration and/or arginase activity in predicting disease recurrence or chance of pregnancy.

### 4.3. Diagnostic Value of Arginase Compared to Other Proposed Non-Invasive Biochemical Markers of Endometriosis

The search for non-invasive markers for endometriosis has proven to be challenging due to the heterogeneity of the disease presentation as well as overlapping autoimmune, endocrine, and inflammatory conditions that affect women with the disease and may influence the sensitivity and specificity of the potential markers.

Although numerous original studies and systematic reviews analysed the potential application of nearly two hundred different molecules as potential markers of endometriosis, and for some of them, a significant association with the presence or absence of endometriosis was found [22,45,46], only single markers meet the criteria of a replacement/screening test. A comparison of test criteria obtained for the best published potential diagnostic markers and Arg-1 is presented in Table 2. It should be noted that in each of the mentioned studies, the number of patients in the endometriosis group was limited, ranging from 47 to 65 patients, compared to 105 patients in this study. Furthermore, the listed studies are only isolated reports or could not be confirmed in further studies.

Serum Arg-1 levels obtained in this study approach the threshold of replacement test criteria (sensitivity ≥ 94% and specificity ≥ 79%) and meet the criteria of both the SnOUT test (high SeNsitivity to rule OUT—i.e., sensitivity ≥ 95% at specificity ≥ 50%, negative result rules out the disease) and SpIN (high SPecificity to rule IN—i.e., sensitivity ≥ 50% at specificity ≥ 95%, positive result confirms the presence of the disease) [12] for the discrimination between endometriosis patients of any stage and age-matched healthy controls.

However, none of the mentioned potential markers, including Arg-1, meets all three criteria for use as a reliable replacement and screening test. Nevertheless, meeting some of the criteria by Arg-1 may provide a basis for further research of their usefulness in this aspect.

### 4.4. Correlations of Arg-1 Concentrations and Serum Arginase Activity with Other Clinical Parameters

Positive correlations between preoperative arginase activity and fasting glucose values observed in this study may partly confirm the relationship between elevated Arg-1 levels, increased arginase activity, and elevated glucose values, and consequently diabetes–through mechanisms inducing reactive oxygen species (ROS) production with subsequent damage to endothelial cells, involving dysfunctions of the nitric oxide synthase (NOS). As a result of these phenomena, there is an accumulation of inflammatory cells (macrophages), the subsequent emergence of foam cells, and activation of a cascade of events leading to progressive dysfunction of vascular endothelium through excessive ROS production [37], excessive expression of Arg-1 causing increased arginase activity, and decreased arginine availability in the microenvironment, with a secondary decrease in NOS activity and nitric oxide (NO) deficiency, excessive production of collagen precursors (due to increased concentrations of arginine breakdown products–ornithine–polyamines, proline), and consequently stiffening of arterial vessels, resulting in a secondary increase in blood pressure and risk of thromboembolic events, common in diabetes [50]. The relationship between glucose values and the concentration of Arg-1 or arginase activity without a diabetic background remains unexplored (lack of available studies).

The positive correlation found in this study between fasting glucose levels and arginase activity, with no correlation with Arg-1 levels, may indirectly indicate a potential role of Arg-2, whose levels were not determined in this study but have been shown in the pathogenesis of diabetes complications such as diabetic nephropathy [51] and diabetic retinopathy [52]. On the other hand, statistically significantly higher fasting glucose levels in group K1 than in the study group may result from the statistically older age of patients in this group (with no results indicating the presence of diabetes).

The variability of arginase isoenzyme concentrations and activity has been studied so far in the context of many pathologies, with most being experimental studies conducted on animal models, while in vivo studies in humans have been conducted to a much lesser extent.

Elevated serum concentrations and activity of arginase isoforms, besides several animal models, were also clinically observed in humans in association with quite common conditions such as diabetes (Arg-2), Alzheimer’s disease (Arg-1/2), multiple sclerosis (Arg-1), ischemic stroke (Arg-1), certain malignancies (Arg-1/2) reviewed in [26]. An opposite phenomenon of decreased serum levels of Arg-1/arginase activity was observed in the case of exacerbation of rheumatoid arthritis, while higher values were observed in the remission phase [53]. Furthermore, elevated levels of arginase isoforms were detected in animal models of quite common and often undiagnosed pathologies like atherosclerosis and arterial and pulmonary hypertension reviewed in [54]. Therefore, it should be considered that these pathologies may significantly and to an undetermined extent disturb the reliability of serum Arg-1 concentrations and arginase activity assessments for endometriosis diagnosis. The absence of determined standards and reference ranges, both in the course of endometriosis and in relation to other diseases, may indeed partially or completely hinder the potential diagnostic value of determining the concentration of Arg-1/arginase activity for the diagnosis of endometriosis.

### 4.5. Perspectives for the Use of Arginase as a Biochemical Marker for Endometriosis

The results obtained in this study do not provide sufficient evidence to consider the determination of serum Arg-1 concentration and make therapeutic decisions. They also do not conclusively indicate the usefulness of Arg-1 levels as a discriminatory biomarker and a sole basis for establishing a diagnosis of endometriosis as well as for monitoring the effectiveness of the treatment process or the completeness of operative treatment for endometriosis. Despite the significant decrease in Arg-1 levels in the study group 48 h after surgical treatment, normalisation of these values (i.e., a decrease to the levels of the control groups, especially of the healthy controls) was not achieved. Moreover, a significant decrease in serum Arg-1 levels after surgery was also observed in the non-endometriosis control group.

The detected elevated levels of serum Arg-1 may indeed indicate its involvement in immunosuppressive and pathomechanisms of endometriosis. Further studies, including immunohistological analysis of histopathological specimens and Arg-1 measurements in peritoneal/cyst fluid of endometriosis patients, as well as correlation with the immunological profile of the patients, are needed to decipher the role of Arg-1 in immune suppression in endometriosis. Additional information would also include the possible determination of the cellular origin of arginase isoenzymes in the microenvironment of endometriosis. Such a study is currently being prepared, with collected histopathological material related to the clinical material presented in this report.

### 4.6. Summary of Prospects and Limitations of Using Arginase in the Diagnosis of Endometriosis

The aforementioned issues and difficulties—including the lack of standards for the methodology of determinations, their reproducibility, ambiguous data regarding the half-lives of arginase isoenzymes, as well as the lack of determined standardised ranges or population reference ranges for both arginase isoenzymes and arginase activity—limit their current use in the diagnosis of endometriosis and other disorders. It would be necessary to conduct large-scale studies in populations of patients with the aforementioned disorders, with appropriately selected control groups, so that a comparison of results between patients with these conditions and healthy individuals is possible.

Developing and implementing a simple, reproducible, sensitive, and specific biochemical test would provide earlier information on whether there are reasons for in-depth clinical and imaging diagnostics and potential treatment (pharmacological or surgical). Another significant area of application for this type of test could also be infertility diagnostics, considering that even half of all cases may result from the presence of endometriosis. The ability to establish a differential diagnosis with greater accuracy would guide healthcare professionals in tailoring appropriate treatment plans for patients.

Implementation of an early non-invasive and easily available diagnostic test would need to be coupled with caution in the interpretation of its results. Similar to the development of biomarkers for other diseases, the potential for earlier diagnosis would need to be balanced with the potential for overdiagnosis and overtreatment. In addition, false-negative results may occur, and the symptoms that led to evaluation should not be invalidated. Repeat testing may be recommended after a negative result if symptoms persist or cannot be explained by any other disease.

The potential clinical applications of Arg 1 analysis in the context of endometriosis may also extend toward treatment strategies. Targeting Arg 1 with specific inhibitors could be explored as a therapeutic approach, as it is already the case in some malignancies [55]. Inhibition of the enzymatic activity of Arg-1 might impede the progression of endometriosis and represent a targeted and tailored approach, offering new possibilities for managing this complex gynaecological condition. In conclusion, further research and clinical trials are essential to validate the potential applications of Arg-1 in clinical practice.

## Figures and Tables

**Table 1:** AUC ROC curve for the Arg-1 concentration comparison between groups GB and GK1/GK2.

ARG-1 Concentration	AUC	SE	AUC Lower 95% CI	AUC Upper 95% CI	*p*
GB vs. GK1	0.848	0.040	0.769	0.926	<0.0001
GB vs. GK2	0.912	0.027	0.859	0.965	<0.0001

GB—study group; GK1—control group 1; GK2—control group 2; AUC—area under curve; SE—AUC standard error, 95% CI—95% confidence interval.

**Table 2:** Comparison of sensitivity/specificity of best potential endometriosis markers, according to the literature, and serum Arg-1 concentrations.

Potential Marker (Cutoff)	Sensitivity	95% CI	Specificity	95% CI	RT	SpIN	SnOUT	Ref.
IL-6 (>12.2 pg/mL)	0.95	0.87–0.99	0.83	0.65–0.94	(+)	n.p	(+)	[47]
Paraoksonase-1 (<141.5 U/L)	0.98	0.89–1.00	0.80	0.64–0.91	(+)	n.p.	(+)	[48]
miR-199a+miR-542-3p (n.p.)	0.97	0.88–1.00	0.88	0.69–0.97	(+)	n.p.	(+)	[49]
Arg-1 (38.15 ng/mL)	0.95	0.89–0.98	0.74	0.60–0.85	(−)	(−)	(+)	
Arg-1 (78.4 ng/mL)	0.61	0.51–0.70	0.96	0.87–0.99	(−)	(+)	(−)	

95% CI—95% confidence interval; n.p.—not provided; (+) meets criteria; (−) does not meet criteria; RT—Replacement Test—sensitivity ≥ 94% and specificity ≥ 79%; SpIN (high SPecificity to rule IN)—sensitivity ≥ 50% with specificity ≥ 95%, positive result indicates disease presence; SnOUT (high SeNsitivity to rule OUT)—sensitivity ≥ 95% with specificity ≥ 50%, negative test excludes the disease [12].

## Data Availability

The data presented in this study are available on request from the corresponding author. The data are not publicly available.

## Supplementary Materials

The following supporting information can be downloaded at https://www.mdpi.com/article/10.3390/jcm13051489/s1, Table S1. Distribution by disease severity and surgery outcome of endometriosis patients in study group GB. Table S2. Follow-up duration in study group following surgery. Table S3. Indications for surgery for patients in control group K1. Table S4. Age parameters in groups B, K1 and K2. Table S5. Fasting blood glucose in study groups. Table S6. Preoperative serum Arg-1 concentrations and arginase activity in study groups. Table S7. Postoperative concentration of Arg-1 and arginase activity in the study group and control group 1, compared with the results of control group 2. Table S8. Comparison of Arg-1 (ng/mL) change (Δ) before and after surgery, between groups GB and K1. Table S9. Comparison of arginase activity (U/L) change (Δ) before and after surgery, between groups B and K1. Table S10. AUC ROC curve for the arginase activity comparison between groups B and K1/K2. Table S11. Correlations of various pre- and postoperative parameters in the study group. Table S12. Correlations of perioperative changes of Arg-1 concentration and arginase activity in the study group. Table S13. Pre- and postoperative Arg-1 concentrations and arginase activities and their perioperative changes, in light of endometriosis recurrence risk following surgery, using the Cox proportional hazards model. Table S14. Observation duration in respect of expected pregnancy in 45 study group infertile patients. Table S15. Pre- and postoperative Arg-1 concentrations and arginase activities and their perioperative changes, in light of chances for pregnancy achievement following surgery, using the Cox proportional hazards model. Figure S1. Age in groups GB, GK1 and GK2. Figure S2. Perioperative change (Δ) in serum arginase activity in groups B and K1. Figure S3. Perioperative change, control group 1. Figure S4. Perioperative change (Δ) in serum arginase activity in control group K1. Figure S5. Correlation between preoperative serum Arg-1 concentration and arginase activity in the study group. Figure S6. Correlation between preoperative serum arginase activity and fasting glucose levels in the study group. Figure S7. Correlation between postoperative serum arginase activity and fasting glucose levels in the study group. Figure S8. Correlations of perioperative changes (Δ) of Arg-1 concentration and arginase activity in the study group.

## Abbreviations

AUC	Area Under the Curve
Arg	Arginase
Arg-1	Arginase-1
Arg-2	Arginase-2
ARGACT	Arginase Activity
ASRM	American Society for Reproductive Medicine
BRCA2	Breast Cancer 2, a gene associated with increased risk of breast cancer
CI	Confidence Interval
DE	Deep Endometriosis
ELISA	Enzyme-Linked Immunosorbent Assay
GB	Group B (Patients with endometriosis)
GK1	Group K1 (Control Group 1)
GK2	Group K2 (Control Group 2)
HLA	Human Leukocyte Antigen
IFN-γ	Interferon-Gamma
IL-2	Interleukin-2
IL-6	Interleukin-6
K1	Control Group 1
K2	Control Group 2
MCP-1	Monocyte Chemoattractant Protein-1
MDSCs	Myeloid-Derived Suppressor Cells
MIF	Macrophage Migration Inhibitory Factor
miR-199a	MicroRNA-199a
miR-542-3p	MicroRNA-542-3p
MRI	Magnetic Resonance Imaging
NOS	Nitric Oxide Synthase
NO	Nitric Oxide
NK	Natural Killer
PON-1	Paraoxonase-1
rASRM	Revised American Society for Reproductive Medicine
ROC	Receiver Operating Characteristic
ROS	Reactive Oxygen Species
SM	Sclerosis Multiplex
SnOUT	Sensitivity to rule OUT
SpIN	Specificity to rule IN
TCR	T-cell Receptor
TNFα	Tumour Necrosis Factor-alpha
TVUS	Transvaginal Ultrasound
USG	Ultrasonography
