# Supplementary material for: Determination of Serum Arginase-1 Concentrations and Serum Arginase Activity for the Non-Invasive Diagnosis of Endometriosis
Source: J Clin Med. 2024 Mar 5;13(5):1489. doi: 10.3390/jcm13051489 (PMC10933979; doi:10.3390/jcm13051489)
Supplement: Supplementary file 1 [file jcm-13-01489-s001.zip › jcm-2829166-SI.pdf]

Journal of
Clinical Medicine

MDPI

Supplementary Materials

Table S1. Distribution by disease severity and surgery outcome of endometriosis patients in study group GB.

**Study group GB**			
**Disease severity**		**surgery**	
Stage I	5 (5%)	optimal	97 (82%)
Stage II	33 (31%)	suboptimal	8 (8%)
Stage III	23 (22%)		
Stage IV	44 (42%)		
Total	105		105

Table S2. Follow-up duration in study group following surgery.

**Study group GB**	**N**	**Mean**	**SD**	**Median**	**Quartile range**	**Range**
Postoperative follow-up time (months)	105	25	8.2	25	19.6 – 30.8	3 – 45

Table S3. Indications for surgery for patients in control group K1.

**Control group K1**	
**Indication for surgery**	
Uterine fibroids	8 (35.4%)
Non-endometrial adnexal cysts	4 (18%)
Diagnostic laparoscopy in cases of pain and infertility	4 (18%)
Ectopic pregnancy	1 (4.6%)
High-grade cervical dysplasia	1 (4.6%)
Endometrial hyperplasia	1 (4.6%)
Douglas pouch cyst	1 (4.6%)
Prophylactic removal of adnexa due to BRCA2 mutation	1 (4.6%)
Pelvic organ prolapse	1 (4.6%)
Total	22

S1. Characteristics of study groups

S1.1. Age

In terms of demographic parameters, a statistically significant age difference was observed between group GB and group K1, as well as between group K2 and group K1 – patients in control group K1 were significantly older in both cases. However, no such difference was found between groups GB and K2. The age characteristics of the study and control groups are presented in Table S4 and presented in Figure S1.

Table S4. Age parameters in groups B, K1 and K2**.**

**Age [years]**	**GB**	**GK1**	**GK2**	**p GB-GK1**	**p GB-GK2**	**p GK1-GK2**
N	105	22	53			
Mean	34.00	37.23	33.96	**0.016**	0.90	**0.043**
SD	5.24	8.13	5.97			
Median	34	39.5	34			
Interquartile range	31 – 37	32 – 42	31 – 37			
Range	21 – 48	19 – 49	18 – 45			

Abbreviations: GB, study group; GK1, control group 1; GK2, control group 2. Statistically significant p values are marked bold (Mann-Whitney U test).

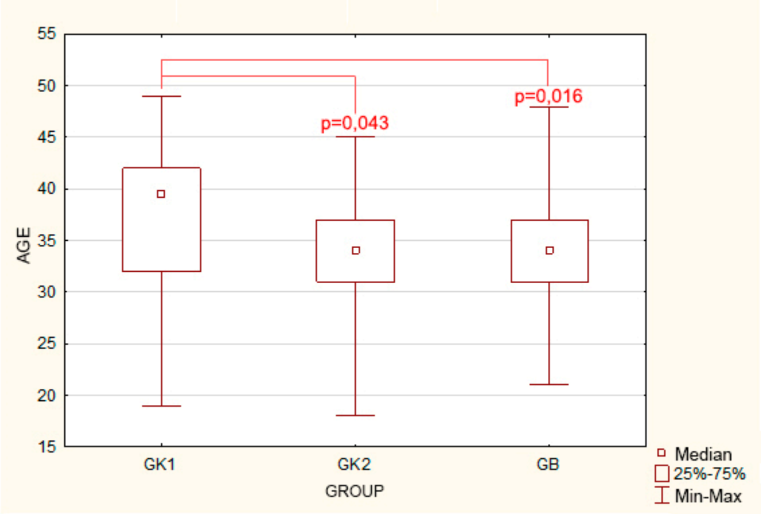

Figure S1. Age in groups GB, GK1 and GK2. Abbreviations: GB, study group; GK1, control group 1; GK2, control group 2. Statistically significant p values are marked (Mann-Whitney U test).

S1.2. Fasting blood glucose

A statistically significant difference in fasting blood glucose was also observed between group GB and group K1. These values were significantly higher in group K1 compared to group BG. However, no significant difference was found between group GB and group K2, or between group K1 and group K2. a Individual levels of fasting blood glucose suggested that a few patients may have pre-diabetes. Given the association of ARG activity and diabetes it would be advisable to adjust for pre-diabetes, however there were not enough patients to perform a subgroup analysis for this respect. Detailed data are presented in Table S5.

Table S5. Fasting blood glucose in study groups**.**

Glycaemia (mg/dL)	GB	N	GK1	N	GK2	N	p GB-GK1	p GB-GK2	p GK1-GK2
Mean ± SD	87.7 ± 7.24	105	92.0 ± 9.17	22	87.4 ± 7.08	53	**0.025**	0.96	0.059
Median	87.0		90.2		87.1				
Interquartile range	83.0 – 92.0		88.5 – 95.9		82.0 – 92.0				
Range	72.0 – 114.1		74.2 – 108.4		73.6 – 105.5				

Abbreviations: GB, study group; GK1, control group 1; GK2, control group 2. Statistically significant p values are marked bold (Mann-Whitney U test).

S1.3. Serum creatinine concentration

No statistically significant differences were found in serum creatinine concentration between group GB and group K1. However, significantly lower creatinine concentrations were observed in group K2 (mean 0.73 mg/dL, median 0.72, interquartile range 0.66 – 0.78, range 0.55 – 1.09) compared to both group GB (mean 0.81 mg/dL, median 0.80, interquartile range 0.7 – 0.9, range 0.6 – 1.1, $p < 0.01$) and K1 (mean 0.81 mg/dL, median 0.84, interquartile range 0.7 – 0.9, range 0.64 – 1.0, $p < 0.05$).

Table S6. Preoperative serum Arg-1 concentrations and arginase activity in study groups.

	GB	N	GK1	N	GK2	N	p GB-GK1	p GB-GK2	p GK1-GK2
**Arg-1 concentration (ng/mL)**									
Mean ± SD	94.1 ± 44.4	105	44.4 ± 23.9	22	32.6 ± 26.5	53	**<0.0001**	**<0.0001**	**0.015**
Median	88.3		30.8		25.6				
Interquartile range	59.3– 114.7		25.1– 69.1		16.8 – 38.8				
Range	13.8 – 212.2		15.1 – 94.4		6.3 – 150.0				
**ARG activity (U/L)**									
Mean ± SD	1.076 ± 0.670	103	0.759 ± 0.312	21	0.733 ± 0.498	53	0.12	**0.003**	0.26
Median	0.906		0.735		0.640				
Interquartile range	0.531 – 1.617		0.569 – 0.928		0.445 – 0.868				
Range	0.103 – 2.778		0.098 – 1.366		0.004 – 2.773				

Abbreviations: ARG, arginase; Arg-1, arginase 1; GB, study group; GK1, control group 1; GK2, control group 2. Statistically significant p values are marked bold (Mann-Whitney U test).

Table S7. Postoperative concentration of Arg-1 and arginase activity in the study group and control group 1, compared with the results of control group 2.

	GB	N	GK1	N	GK2	N	p GB-GK1	p GB-GK2	p GK1-GK2
**Arg-1 concentration (ng/mL)**									
Mean ± SD	75.0 ± 46.3	93	28.8 ± 20.5	14	32.6 ± 26.5	53	**0.0005**	**<0.0001**	0.59
Median	62.7		24.1		25.6				
Interquartile range	45.5– 89.6		12.4– 34.7		16.8 – 38.8				
Range	15.1 – 299.0		5.35 – 71.4		6.3 – 150.0				
**ARG activity (U/L)**									
Mean ± SD	1.041 ± 0.773	91	0.603 ± 0.578	13	0.733 ± 0.498	53	**0.041**	**0.044**	0.19
Median	0.915		0.484		0.640				
Interquartile range	0.427 – 1.519		0.201 – 0.743		0.445 – 0.868				
Range	0.000 – 3.317		0.097 – 2.297		0.004 – 2.773				

Abbreviations: ARG, arginase; Arg-1, arginase 1; GB, study group; GK1, control group 1; GK2, control group 2. Statistically significant p values are marked bold (Mann-Whitney U test).

Table S8. Comparison of Arg-1 (ng/mL) change (Δ) before and after surgery, between groups GB and K1.

	GB	n	GK1	n	P
**Δ Arg-1**					
Mean ± SD	-19.1 ± 63.5	93	-13.7 ± 11.3	14	**0.44** [a]
Median	-20.2		-16.3		
Interquartile range	-42.3 to +1.94		-21.0 to -8.5		
Range	-158.3 to +256.8		-33.1 to +9.6		
p for Δ Arg1	**<0.0001** [b]		**0.0043** [b]		

Abbreviations: Arg-1, arginase 1; GB, study group; GK1, control group 1; GK2, control group 2. Statistically significant p values are marked bold, Mann-Whitney U test ([a]) or Wilcoxon pair signed-rank test ([b]).

Table S9. Comparison of arginase activity (U/L) change (Δ) before and after surgery, between groups B and K1.

	GB	N	GK1	N	P
**ARG activity Δ**					
Mean ± SD	-0.053 ± 0.674	91	-0.106 ± 0.548	13	0.5 [a]
Median	-0.025		-0.133		
Interquartile range	-0.429 to 0.301		-0.293 to -0.004		
Range	-1.428 to 1.669		-0.779 to 1.403		
**p for ARG activity Δ**	0.35 [b]		0.13 [b]		

Abbreviations: ARG, arginase ; GB, study group; GK1, control group 1; GK2, control group 2. p values calculated using the Mann-Whitney U test ([a]) or Wilcoxon pair signed-rank test ([b]).

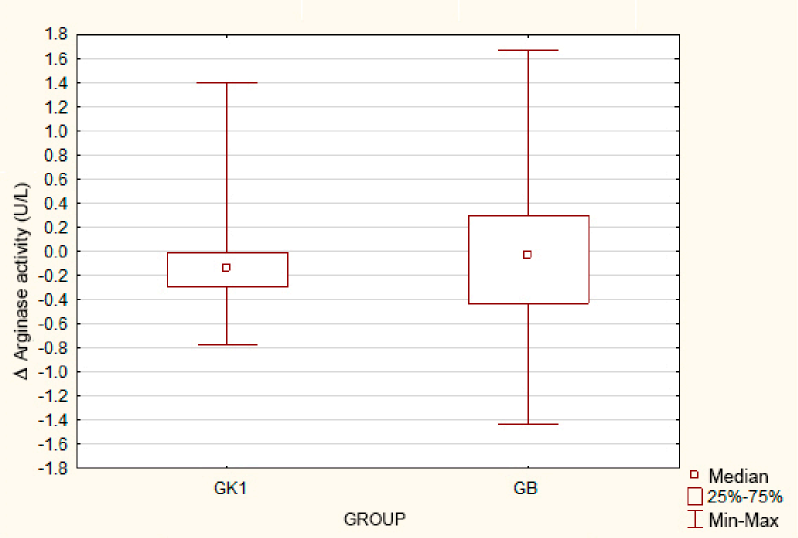

Figure S2. Perioperative change (Δ) in serum arginase activity in groups B and K1**.** Abbreviations: GB, study group; GK1, control group 1.

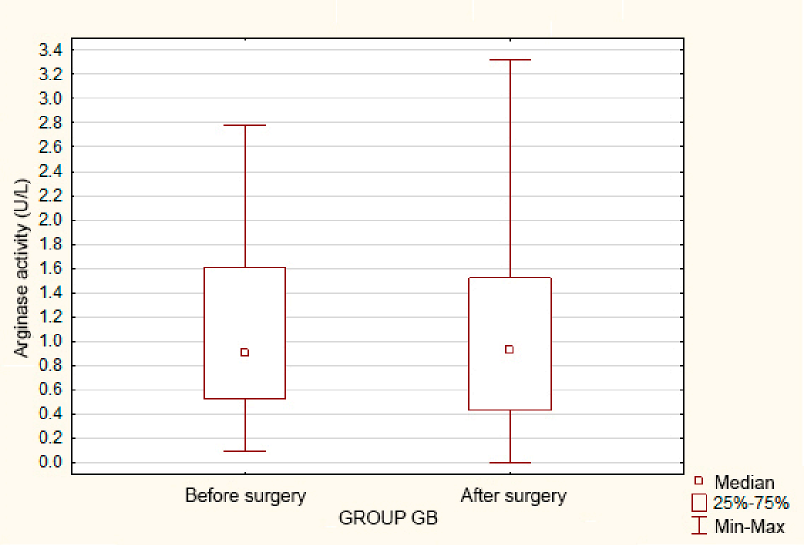

Figure S3. Perioperative change, control group 1.

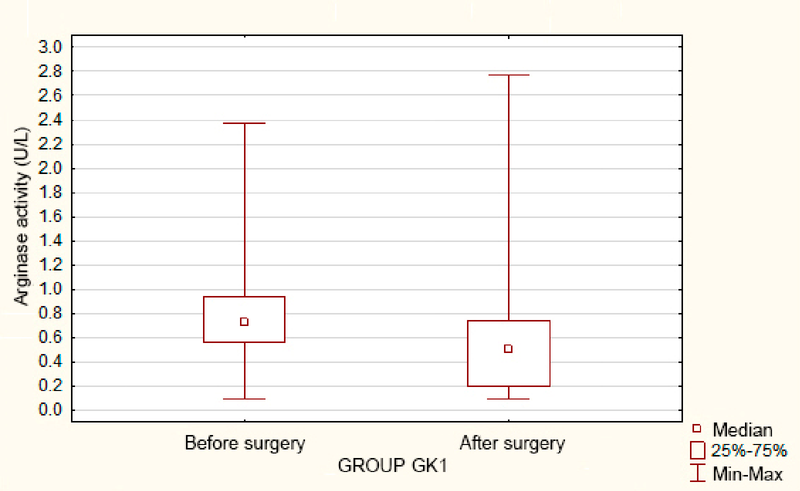

Figure S4. Perioperative change (Δ) in serum arginase activity in control group K1.

Table S10. AUC ROC curve for the arginase activity comparison between groups B and K1/K2.

**Arginase activity**	**AUC**	**SE**	**AUC lower 95% CI**	**AUC upper 95% CI**	**p**
GB vs GK1	0.608	0.054	0.501	0.715	0.047
GB vs GK2	0.646	0.045	0.559	0.734	0.001

Abbreviations: GB, study group; GK1, control group 1; GK2, control group 2; AUC, area under curve; SE, AUC standard error, 95% CI, 95% confidence interval.

Table S11. Correlations of various pre- and postoperative parameters in the study group.

	**Arg-1 level preop.**		**N**	**Arg-1 level postop.**		**N**	**Arg activity preop.**		**N**	**Arg activity postop.**		**N**
	**R**	**p**		**R**	**p**		**R**	**p**		**R**	**P**	
Age	0.04	0.71	105	**0.20**	**0.048**	93	0.01	0.88	103	0.06	0.59	91
Grading (ASRM)	-0.02	0.83	105	0.01	0.94	93	**0.22**	**0.029**	103	0.09	0.39	91
Fasting glucose	-0.06	0.51	105	-0.07	0.47	93	**0.27**	**0.006**	103	**0.29**	**0.005**	91
Arg-1 level preop.				0.18	0.08	93	**0.29**	**0.003**	103			
Arg-1 level postop.	0.18	0.08	93							0.19	0.08	91
ARG activity preop.	**0.29**	**0.003**	103							**0.59**	**<0.001**	91
ARG activity postop.				0.19	0.08	91	**0.59**	**<0.001**	91			

Abbreviations: Arg, arginase; Arg-1, arginase 1. R – Spearman rank correlation coefficient; p – statistical significance level (significant values are marked bold).

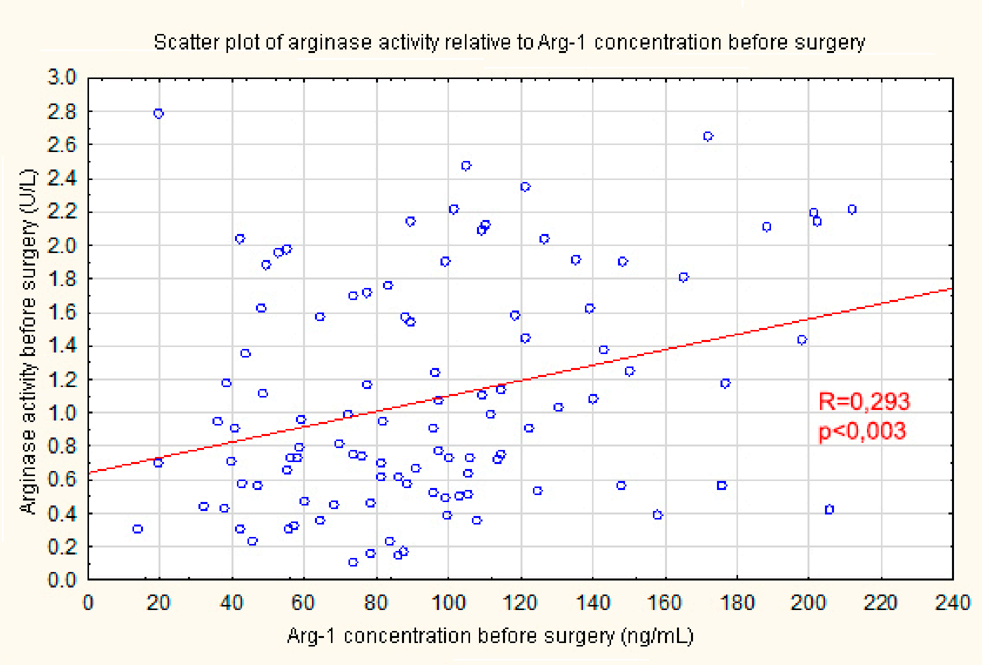

Figure S5. Correlation between preoperative serum Arg-1 concentration and arginase activity in the study group. Abbreviations: Arg-1, arginase 1. R –Spearman rank correlation test.

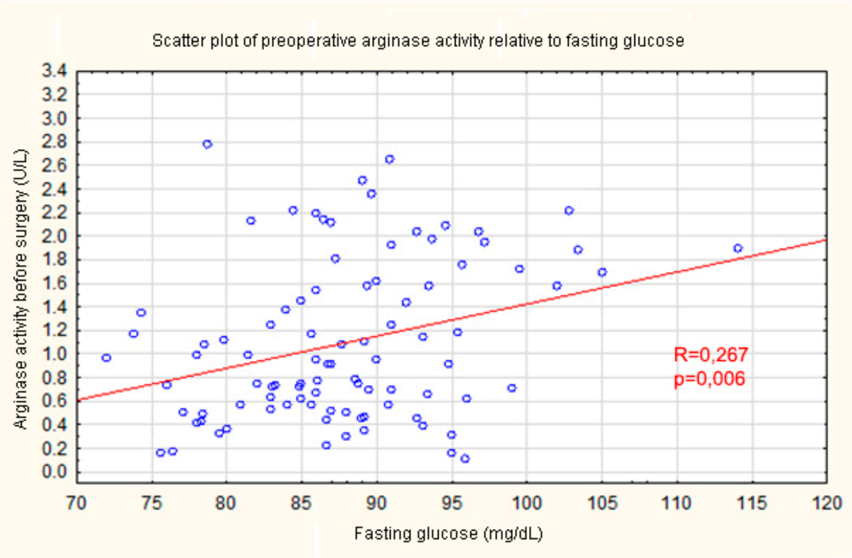

Figure S6. Correlation between preoperative serum arginase activity and fasting glucose levels in the study group. R –Spearman rank correlation test.

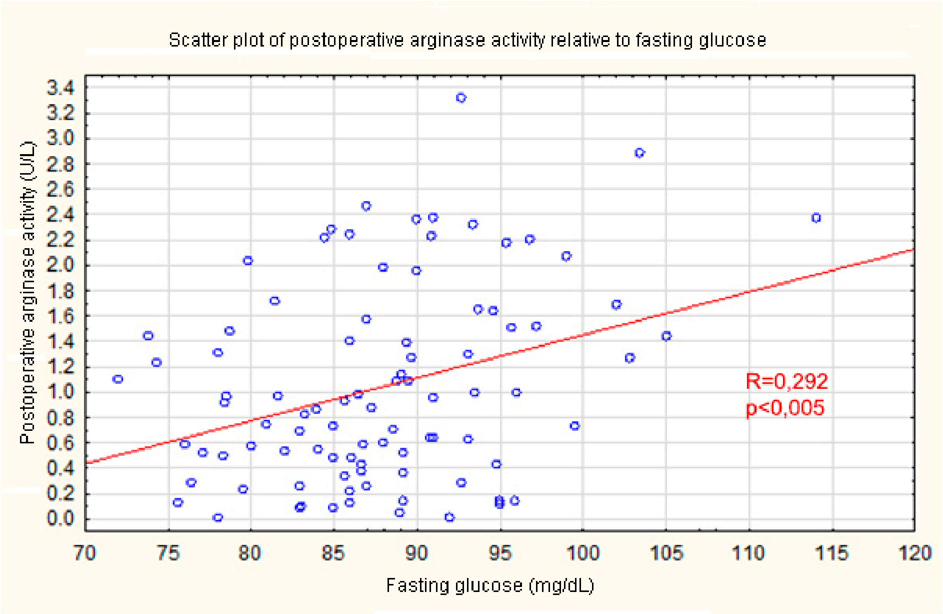

Figure S7. Correlation between postoperative serum arginase activity and fasting glucose levels in the study group. R –Spearman rank correlation test.

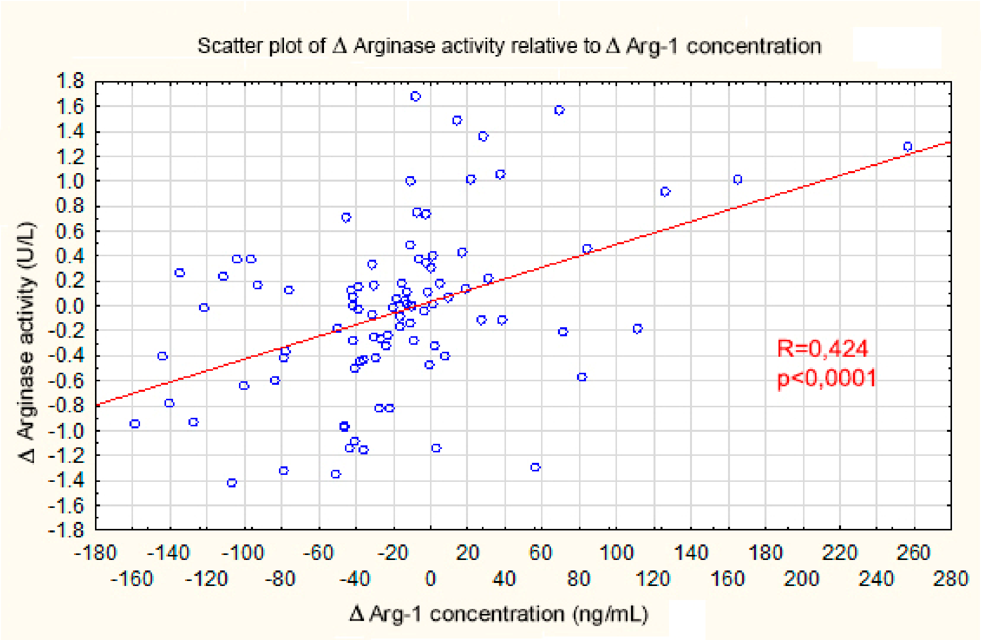

Figure S8. Correlations of perioperative changes (Δ) of Arg-1 concentration and arginase activity in the study group. R – Spearman rank correlation test. Abbreviations: Arg-1, arginase 1.

Table S12. Correlations of perioperative changes of Arg-1 concentration and arginase activity in the study group.

	**Δ Arg-1**		**N**	**Δ ARG_{act}**		**N**
	**R**	**p**		**R**	**p**	
Δ Arg-1				**0.42**	**<0.0001**	91
**Δ ARG_{act}**	**0.42**	**<0.0001**	91			
Age	0.14	0.18	93	0.07	0.49	91
Grading (ASRM)	0.06	0.59	93	-0.05	0.62	91
Fasting glucose	0.00	0.97	93	0.04	0.73	91

Abbreviations: Arg-1, arginase 1; Δ Arg-1, perioperative arginase 1 concentration change (pre-postoperatively); Δ ARG_{act}, arginase activity change (pre-postoperatively); R - Spearman rank correlation test. Statistical significance level, with a significance threshold of $p<0.05$ (bold).

Table S13. Pre- and postoperative Arg-1 concentrations and arginase activities and their perioperative changes, in light of endometriosis recurrence risk following surgery, using the Cox proportional hazards model.

**Recurrence predictor**	**HR**	**95% CI**	**p**
Preoperative Arg-1 concentration	1.006	0.995 – 1.018	0.28
Postoperative Arg-1 concentration	1.008	0.998 – 1.019	0.13
Preoperative arginase activity	1.088	0.509 – 2.326	0.83
Postoperative arginase activity	1.445	0.762 – 2.738	0.26
Δ Arg-1	1.000	0.991 – 1.010	0.93
**Δ ARG_{act}**	1.527	0.716 – 3.269	0.27

Abbreviations: Arg-1, arginase 1; Δ Arg-1, perioperative arginase 1 concentration change; Δ ARG_{act}, perioperative arginase activity change; HR, Hazard Ratio (relative recurrence hazard in the Cox proportional hazards model); 95% CI, 95% confidence interval.

Table S14. Observation duration in respect of expected pregnancy in 45 study group infertile patients.

**Study group**	**Mean**	**SD**	**Median**	**Interquartile range**	**Range**
Observation duration until pregnancy/negative observation (months)	11.9	10.9	11.5	4.5 – 23.5	1 – 39

The table presents parameters related to the time of postoperative observation until pregnancy in the group of patients with infertility. The observation time for achieving pregnancy applied exclusively to patients with infertility and covered the period from surgery until pregnancy was achieved or until the last negative observation in this regard.

Table S15. Pre- and postoperative Arg-1 concentrations and arginase activities and their perioperative changes, in light of chances for pregnancy achievement following surgery, using the Cox proportional hazards model.

Pregnancy predictor	HR	95% CI	p
Preoperative Arg-1 concentration	1.000	0.991 – 1.009	0.98
Postoperative Arg-1 concentration	0.999	0.987 – 1.011	0.84
Preoperative arginase activity	1.118	0.654 – 1.910	0.68
Postoperative arginase activity	1.193	0.682 – 2.086	0.54
Δ Arg-1	1.000	0.992 – 1.008	0.98
**Δ ARG_{act}**	1.059	0.414 – 2.706	0.90

Abbreviations: Arg-1, arginase 1; Δ Arg-1, perioperative arginase 1 concentration change; Δ ARG_{act}, perioperative arginase activity change; HR, Hazard Ratio (relative recurrence hazard in the Cox proportional hazards model); 95% CI, 95% confidence interval.
